# The interaction of Wnt signaling members with growth factors in cultured granulosa cells

**DOI:** 10.1590/1984-3143-AR2019-0106

**Published:** 2020-06-17

**Authors:** Filiz Tepekoy, Gokhan Akkoyunlu

**Affiliations:** 1 Department of Histology and Embryology, Faculty of Medicine, Altinbas University, Istanbul, Turkey; 2 Department of Histology and Embryology, Faculty of Medicine, Akdeniz University, Antalya, Turkey

**Keywords:** EGF, FGF, FZD, IGF, WNT

## Abstract

Wnt family members have recently been distinguished in the adult ovary with potential roles in ovarian function. Though particular growth factors interact with Wnt signaling members in extraovarian cell types, it is unclear whether this interaction is applicable in the granulosa cells. Therefore, the current study aimed to determine the effect of insulin-like growth factor-1 (IGF-I), epidermal growth factor (EGF) and basic fibroblast growth factor (FGF-β) on Wnt ligands WNT2 and WNT4 and Wnt receptor Frizzled-4 (FZD4) protein levels in cultured mouse granulosa cells. Granulosa cells were isolated from antral follicles of adult Balb/C mice and cultured for 24 hours in the presence of 100 ng/mL of IGF-I, or EGF or FGF-β. WNT2, WNT4 and FZD4 protein levels were evaluated through western blotting after the culture process. IGF-I treated granulosa cells had significantly the highest level of WNT2 and WNT4 as well as FZD4 when compared to FGF-β and EGF groups. FGF-β group had a significantly higher level of WNT2, WNT4 and FZD4 expression when compared to EGF group. FZD4 expression was at the highest level in the IGF-I group and this difference was statistically significant for all groups including uncultured cells and vehicle group. In addition, FGF-β was shown to positively affect the adhesion of granulosa cells. This study demonstrates that IGF-I, FGF-β and EGF have differential effects on the expressions of WNT2, WNT4, and FZD4 in cultured mouse granulosa cells, suggesting that particular growth factors related to ovarian function might conduct their roles in the ovary through Wnt signaling.

## Introduction

Wnt signaling is one of the key pathways that regulate critical processes during embryonic development (Van Amerongen and Nusse, 2009). The Wnt signaling is composed of hydrophobic Wnt ligands that are found in the extracellular matrix, G-protein coupled transmembrane frizzled receptors (FZD) and low density lipoprotein receptor-related protein (LRP) co-receptors and an effector protein, β-catenin (CTNNB1) residing in the cytoplasm or in the nucleus depending on the activation status of the signaling (Tepekoy et al., 2015).

Wnt signaling acts through three different pathways: the canonical Wnt/β-catenin cascade, the non-canonical planar cell polarity pathway, and the Wnt/Ca^2^+ pathway (Niehrs, 2012). The canonical Wnt signaling cascade transmits its signals through the effector protein β-catenin and regulates target gene expression (Alok et al., 2017). When the signal is not activated, β-catenin is localized in the cytoplasm and it is associated with a protein complex composed of adenomatous polyposis coli (APC), glycogen synthase kinase (GSK)-3B and Axin. This protein complex enables the phosphorylation and thereby degradation of cytoplasmic β-catenin. The canonical signal is activated when a Wnt ligand in the extracellular space, associated with the cell membrane binds to a transmembrane FZD receptor which cooperates with a member of the LRP family (Janda et al., 2012; Logan and Nusse, 2004). Upon the activation of the signal, the protein complex of APC, (GSK)-3B and Axin is disrupted and dissolution of this complex culminates in the prevention of β-catenin degradation (Fiedler et al., 2011). β-catenin accumulates in the cytoplasm and is translocated to the nucleus where it controls transcriptional regulation through binding T-cell factor/lymphoid enhancer binding protein (TCF/LEF) (Gordon and Nusse, 2006; Schuijers et al., 2014).

In addition to their roles in embryonic gonad development (Chassot et al., 2012), Wnt family members have recently been distinguished in the adult ovary (Boyer et al., 2010). The folliculogenesis process in the adult ovary has been discovered to be affected by Wnt signaling (Abedini et al., 2016; Boyer et al., 2010; Wang et al., 2010a). *Wnt4* ablation in mouse granulosa cells is associated with impaired antral follicle development and subfertility (Boyer et al., 2010). Though the depletion of *Ctnnb1* in granulosa cells do not cause ovarian functional defect (Parakh et al., 2006), β-catenin is related to FSH-induced follicular growth and inhibition of the follicle atresia (Fan et al., 2010). On the other hand, healthy development of the ovarian follicles is regulated through Wnt signaling (Li et al., 2014).

Wnt signaling members are known to regulate ovarian follicle response to the gonadotropins (Boyer et al., 2010; Lapointe and Boerboom, 2011). It has been revealed that FSH regulates WNT2 expression in primary cultures of bovine granulosa cells (Castanon et al., 2012). Dominant follicle selection has been reported to be related to Wnt signaling under the control of FSH (Gupta et al., 2014). Wnt receptors FZD4 and FZD1 are also induced by the LH surge in the rodent ovary (Hsieh et al., 2003). Wnt signaling inhibitor sFRP4 expression was decreased when granulosa cells were stimulated by LH/hCG in vivo or in vitro (Maman et al., 2011).

Reproductive hormones regulate ovarian functions through the activation of particular growth factors (El-Hayek et al., 2014; Li et al., 2019). Epidermal growth factor (EGF) signaling was discovered to be a downstream target of both LH (Ashkenazi et al., 2005) and FSH (El-Hayek et al., 2014) to regulate physiological processes in the ovary. Ovarian follicle growth and differentiation as well as steroidogenesis are associated with EGF (Mondschein and Schomberg, 1981; Schomberg et al., 1983). For granulosa cell proliferation, induction of DNA synthesis through phosphorylation of MAPK3/1 was reported to be achieved by tyrosine kinase activity of EGFR that is activated by EGF (Yang and Roy, 2006).

FGF signaling regulates ovarian follicle development (Price, 2016). FGFs are known to stimulate mitosis in cultured granulosa cells (Gospodarowicz and Bialecki, 1979) and inhibit apoptosis in rat granulosa cells (Peluso and Pappalardo, 1999). FGFs are also related to activation of primordial follicle development in rat and human ovaries (Garor et al., 2009; Kezele et al., 2005; Nilsson et al., 2001). FGF is known to interact with FSH for activation of in vitro cultured ovarian follicles (Matos et al., 2007). FGF-β expression was suggested to be altered by LH in bovine ovarian follicles (Berisha et al., 2006).

Members of the insulin-like growth factor (IGF) family were reported to have functions in the development of preantral to preovulatory follicles and follicular atresia (Hastie and Haresign, 2006; Zhao et al., 2001). When added to culture media, IGF-I (1-100 mg/L) stimulated the in vitro development of rat secondary follicles through activation follicular cell proliferation as well as differentiation (Zhao et al., 2001). In preantral follicles, FSH receptor expression was reported to be associated with IGF-I (Silva et al., 2009; Zhou et al., 1997). Preovulatory LH surge was found to alter the phosphorylation of IGF1R and activate its downstream targets (Schuermann et al., 2018).

Since growth factors and also Wnt signaling members are under the control of gonadotropins, the actions of gonadotropins on Wnt signaling members might be through these growth factors. Thus, the aim of the current study was to investigate the effects of growth factors IGF-I, FGF-β and EGF on Wnt ligands WNT2, WNT4 and Frizzled receptor FZD4 protein expression levels in cultured mouse granulosa cells in order to determine the interaction between growth factors and Wnt signaling members.

## Material and methods

### Animals and granulosa cell culture

Female (n=18) Balb/C intact mice supplied by Animal Care and Usage Committee of Akdeniz University were maintained under standard laboratory conditions (21±1 °C; ambient temperature; controlled light/dark conditions, 14L: 10D) and were given food and water ad libitum. The experimental protocol was approved by the Animal Ethics Committee of Akdeniz University, Turkey (2011.11.01).

Intraperitoneal 5IU PMSG injection was applied to 6 weeks old female mice and ovaries were harvested after 48 hours of injection. Antral follicles of 12 ovaries from 6 animals were punctured in Ham’s F12 media under stereomicroscope after incubation with EGTA solution (6mM) for 5 min and Sucrose solution (0.5M) for 15 minutes. The cells were strained with 40 µm cell strainer and centrifuged at 500 x g for 10 min. After removal of the supernatant, the cells were re-suspended in Ham’s F12 media supplemented with FBS (10%), ITS (0.2%) and penicillin-streptomycin (1%). This cell suspension was divided into 5 groups. 1) Uncultured granulosa cell group: Laemli solution was added (1:1) to the cell suspension for lysis of the cells. This cell suspension was stored in Laemli solution in -80 °C freezer for western blot analysis without any cell culture application; 2) Vehicle group: The cells were placed in the 6 well plate for 24h of culture in the Ham’s F12 media with above-mentioned supplements and only PBS was added to the culture media; 3) IGF-I group: IGF-I (100 ng/mL) was added in the culture media of the granulosa cells; 4) FGF-β group: FGF-β (100 ng/mL) was added in the culture media of the granulosa cells; 5) EGF group: EGF (100 ng/mL) was added in the culture media of the granulosa cells. All the cells in the culture groups were incubated for 24h at 37 °C and 5% CO_2_. After the culture, the cells were removed from the plates, treated with trypsin and centrifuged at 500 x g for 10 min after the addition of Ham’s F12 media supplemented with 10% FBS. After removal of the supernatant, the cells were suspended in Laemli solution for lysis. The cell lysates were preserved in -80 °C freezer until they were processed for the western blotting. These experiments were repeated three times with inclusion of 6 animals in each replicate.

### SDS polyacrylamide gel electrophoresis and western blotting

In order to perform immunoblot analysis of WNT2, WNT4, and FZD4; samples were subjected to SDS polyacrylamide gel electrophoresis and then were transferred onto nitrocellulose membranes (Amersham Pharmacia, Piscataway, NJ, USA) in a buffer containing 0.2 mol/l glycine, 25 mMTris and 20% methanol overnight. The successful transfer was confirmed by Ponceau S (Sigma-Aldrich Co. LLC, Steinheim, Germany) staining of the blots. The membranes were blocked for 1 h with 5% non-fat dry milk (BioRad, Hercules, CA, USA) and 0.1% Tween 20 (Sigma-Aldrich Co. LLC, Steinheim, Germany) in 0.14 mol/l Tris-buffered saline (TBS) pH:7.2–7.4 at 4 °C. Blotting membranes were incubated overnight at 4 °C with rabbit polyclonal antibodies WNT2 (1:1000), WNT4 (1:1000) and FZD4 (1:500) (Santa Cruz, CA, USA) at the dilutions specified in the parenthesis. After washing steps, the membranes were further incubated with goat anti-rabbit IgG horseradish peroxidase conjugate (BioRad, Hercules, CA, USA) diluted 1:3000 for 1 h at room temperature. Immunolabeling was visualized using the chemiluminescence based SuperSignal CL HRP Substrate System (Pierce, Rockford, IL, USA) and the membranes were exposed to Hyperfilm (Amersham Pharmacia). GAPDH antibody (1:5000 dilution) (Abcam, Cambridge, UK) was used as an internal control for each blotting in order to confirm the equal loading of the samples. The bands were quantified using NIH image analysis software (ImageJ Version 1.36b, National Institutes of Health, Bethesda, MD, USA).

### Statistical analysis

Western blotting data from ImageJ were analyzed with non-parametric ANOVA on ranks (Kruskal–Wallis test) and parametric One-way ANOVA, Holm Sidak method. The values were presented as mean ± SEM. Statistical calculations were performed using Sigma Stat for Windows, version 3.0 (Jandel Scientific Corp. San Rafael, CA, USA). Statistical significance was defined as P <0.05.

## Results

**Figure 1:**
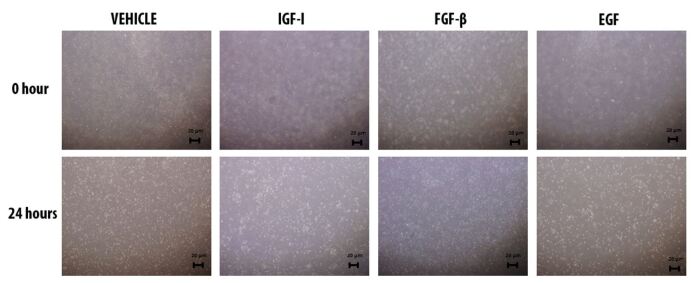
Cultured mouse granulosa cells from antral ovarian follicles. Vehicle group: Ham’s F12 medium+PBS. IGF-I group: Ham’s F12+ 100 ng/mL IGF-I. FGF-β group: Ham’s F12+ 100 ng/mL FGF-β. EGF group: Ham’s F12+ 100 ng/mL EGF.

**Figure 2:**
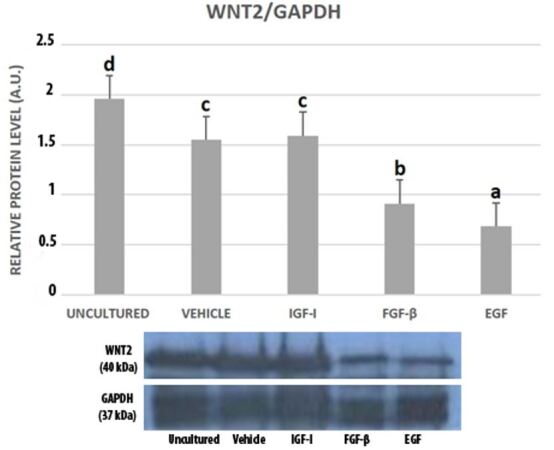
Western blot bands and graphics of mathematical values of ImageJ evaluations of WNT2 protein expressions in uncultured and cultured mouse granulosa cells in the presence of IGF-I (100 ng/mL), FGF-β (100 ng/mL) and EGF (100 ng/mL). Different letters mark statistical significance (*P*< 0.05) (One-way ANOVA, Holm Sidak method).

The granulosa cells subjected to a culture period of 24h with addition of different growth factors were observed (Figure 1) before detection of WNT signaling members through the western blotting. According to the western blotting analysis, WNT2, WNT4 and FZD4 expressions were present in all groups of cultured granulosa cells as well as uncultured cells. The WNT2 expression level was significantly the highest in uncultured granulosa cells. Vehicle and IGF-I group of cultured granulosa cells had higher WNT2 expression level when compared to FGF-β and EGF groups. Though WNT2 protein level in the IGF-I group was higher than the vehicle group, this difference was not statistically significant. FGF- β treated granulosa cells had a higher WNT2 expression level when compared to EGF treated granulosa cells (Figure 2).

**Figure 3:**
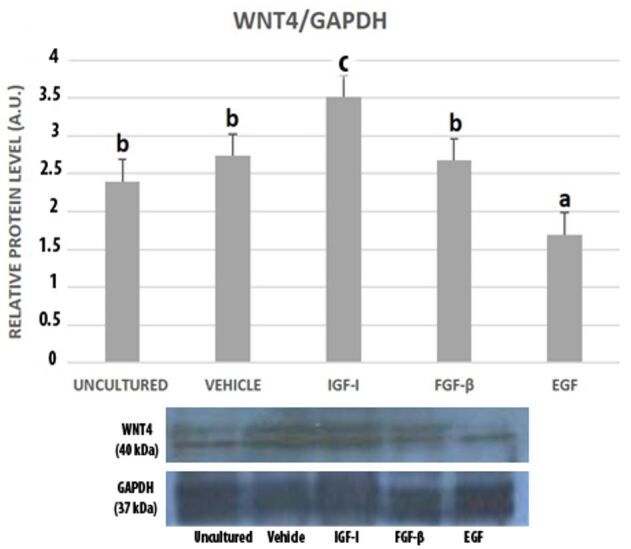
Western blot bands and graphics of mathematical values of ImageJ evaluations of WNT4 protein expressions in uncultured and cultured mouse granulosa cells in the presence of IGF-I (100 ng/mL), FGF-β (100 ng/mL) and EGF (100 ng/mL). Different letters mark statistical significance (*P*< 0.05) (One-way ANOVA, Holm Sidak method).

WNT4 expression was significantly the highest in IGF-I treated granulosa cells. EGF group had the lowest WNT4 level when compared to both uncultured and cultured groups. There wasn’t a statistically significant difference among uncultured, vehicle and FGF-β groups (Figure 3).

**Figure 4:**
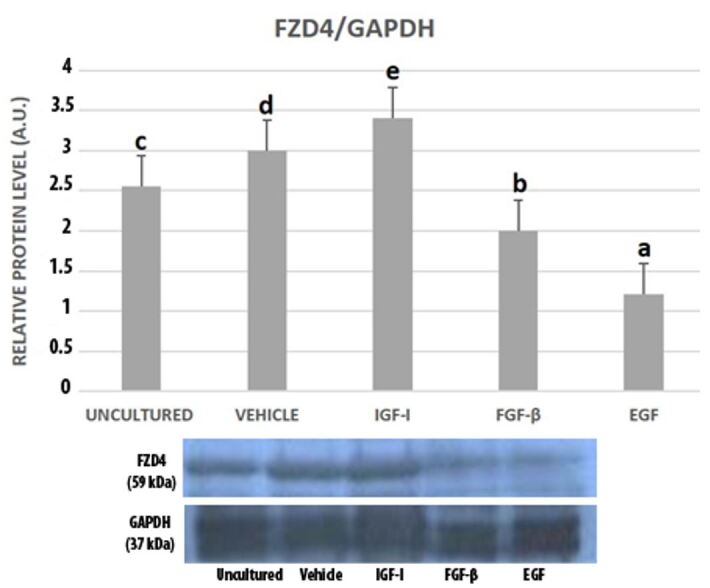
Western blot bands and graphics of mathematical values of ImageJ evaluations of FZD4 protein expressions in uncultured and cultured mouse granulosa cells in the presence of IGF-I (100 ng/mL), FGF-β (100 ng/mL) and EGF (100 ng/mL). Different letters mark statistical significance (*P*< 0.05) (One-way ANOVA, Holm Sidak method).

FZD4 expression was at the highest level in IGF-I group among all groups and this difference was statistically significant. IGF-I group was followed by vehicle group and uncultured granulosa cells respectively in terms of FZD4 levels. FGF-β treated granulosa cells had a lower FZD4 level when compared to IGF-I, vehicle and uncultured groups. EGF treated cells had significantly the lowest FZD4 level among all groups (Figure 4).

## Discussion

This study demonstrates that IGF-I, FGF-β and EGF have differential effects on the expressions of Wnt ligands WNT2 and WNT4 as well as Frizzled receptor FZD4 in cultured mouse granulosa cells.

The folliculogenesis process in the ovary is under the control of hormonal regulation and ovarian factors (Hsueh et al., 2015). The pituitary gonadotropins FSH and LH are the leading actors of hormonal regulation during folliculogenesis and the expression and activation of Wnt signaling members in the ovary are known to be affected by these hormones (Conti et al., 2006). However, in ovarian cells, the mechanism of activation of Wnt ligands and receptors still remain unclear.

Both β-catenin dependent canonical and β-catenin independent non-canonical Wnt signaling pathways take part in the events regarding the proper functioning of the female reproductive tract (Miller et al., 1998). In recent studies, Wnt pathway was reported to take part in ovarian function, regulating structural properties (Wang et al., 2013) and hormone synthesis (Qiao et al., 2017; Stapp et al., 2014) as well as regulation of hormone functions (Castanon et al., 2012) in granulosa cells.

In the current study, among three growth factors applied, remarkably IGF-I was found to provide higher expression levels of Wnt signaling members in granulosa cells and these levels were closer to uncultured and vehicle groups. IGF-I was reported to induce tyrosin phosphorylation of β-catenin resulting in detachment of β-catenin from E-cadherin complexes and improvement in cytoplasmic β-catenin levels (Playford et al., 2000). In addition to its roles in β-catenin stabilization, IGF-I was found to regulate the localization and transcriptional activity of β-catenin (Playford et al., 2000).

Though it has been found that IGF signaling is associated with Wnt signaling in particular cell types such as spermatogonial (Safian et al., 2018), satellite (Siegle et al., 2018), hepatoma cells (Desbois-Mouthon et al., 2001) and chondrocytes (Wang et al., 2010b), the relation between Wnt signaling and IGF-I has not been identified in granulosa cells. In granulosa cells, IGF-I and IGF1R were previously shown to regulate FSH dependent events (Baumgarten et al., 2014; Zhou et al., 2013). In cumulus cells FSH-regulated genes were shown to be associated with IGF1R activity (Stocco et al., 2017).

Since FSH was found to suppress Wnt signaling antagonist SFRP4, it was proposed in the previous studies that FSH prevented early luteinization of GCs by inhibiting SFRP4 (Stocco et al., 2017). It was also observed that FSH caused decreased expression of disheveled binding antagonist of β-catenin 1 (DACT1), and therefore it was suggested that FSH indirectly stimulated the WNT pathway (Stocco et al., 2017). These suggestions are consistent with the findings of the current study which shows the contribution of IGF-I in the increased expression of Wnt signaling members in granulosa cells. One of the targets of this interaction between IGF-I and Wnt signaling might be Indian Hedgehog (IHH) signaling which is a possible regulator of folliculogenesis, as in GC treated with IGF-I, IHH mRNA was found to be increased by WNT3A (Aad et al., 2012). Though in the recent studies it has been found that IGF-I regulates FSH dependent β-catenin activation through AKT signaling in bovine granulosa cells (Gomez et al., 2018), according to our results, we suggest that WNT ligands and receptors might also be activated through IGF-I.

In addition to IGF-I, FGF-β (Holnthoner et al., 2002) and EGF (Cheon et al., 2004) were also reported to activate the β-catenin pathway in endothelial cells and fibroblasts. However, the relation between these growth factors and Wnt signaling has not yet been revealed in ovarian cells.

EGF was reported to regulate liver cell proliferation through the interaction between MAPK/ERK signaling and Wnt signaling under the control of EGF (Jin et al., 2011). WNT1 and WNT5a were reported to induce MAPK pathway through EGFR in HC11 breast gland epithelial cells (Musgrove, 2004). In U87 glioblastoma cell line, β-catenin transactivation was found to be achieved via EGFR (Yang et al., 2011). In a recent study including epithelial follicle stem cells, Wnt signaling was found to be relevant to the EGFR pathway during self-renewal of these cells (Kim-Yip and Nystul, 2018).

In mouse corpus luteum, Wnt signaling was found to activate the EGFR-ERK pathway (Pan et al., 2014), suggesting an interaction between these pathways in the ovarian cells. However, in granulosa cells a relation between Wnt signaling and EGF pathway has not been reported in previous studies. Our results revealed that EGF treated granulosa cells had the lowest level of WNT2, WNT4 and FZD4 expression, suggesting an inhibitory effect of this growth factor on Wnt signaling in granulosa cells.

Although the interaction of WNT and FGF signaling during Xenopus gastrulation (Kjolby et al., 2019) and mouse trachea development (Hou et al., 2019) was revealed, the relationship between these pathways were not specified in granulosa cells during folliculogenesis. During epithelial branching morphogenesis, FGF was found to act as an inhibitor of Wnt/β-catenin signaling (Patel et al., 2011). In the current study, decreased levels of WNT2, WNT4 and FZD4 in granulosa cells cultured in the presence of EGF and FGF-β might suggest that these growth factors inhibit the expression of Wnt signaling members in the adult mouse ovary. As a result of inhibition of Wnt ligands and receptors, these growth factors might also inactivate cytoplasmic β-catenin, resulting in increased levels of β-catenin related to E-cadherin and improve E-cadherin mediated cell adhesion.

## Conclusion

The expressions of WNT ligand WNT4 as well as Frizzled receptor FZD4 in the current study were significantly increased by IGF-I in cultured granulosa cells compared to the vehicle groups. Thus, particular members of the Wnt signaling might be regulated mainly by IGF-I. In order to clearly reveal the mechanism of action of IGF-I and other growth factors on Wnt signaling in granulosa cells, functional studies regarding possible upstream and downstream regulators must be conducted.
